# A Regioselective Synthesis of *E*-Guggulsterone

**DOI:** 10.3390/molecules16054165

**Published:** 2011-05-20

**Authors:** Jungyeob Ham, Jungwook Chin, Heonjoong Kang

**Affiliations:** 1 Korea Institute of Science and Technology, 290 Daejeon-dong, Gangneung 210-340, Korea; Email: ham0606@kist.re.kr (J.H.); 2 Center for Marine Natural Products and Drug Discovery, School of Earth and Environmental Sciences, Seoul National University, NS-80, Seoul 151-747, Korea; 3 Research Institute of Oceanography, Seoul National University, NS-80, Seoul 151-741, Korea

**Keywords:** guggulsterone, inflammatory bowel diseases, regioselective synthesis, oppenhauer oxidation

## Abstract

We have successfully prepared *E*-guggulsterone from 16,17-epoxy-pregnenolone in 84% yield over two steps via a hydrazine reduction and Oppenhauer oxidation. Additionally, isomerization was induced by heat, light (*hν*) and acid catalysis to convert *E*- guggulsterone into the corresponding *Z* isomer.

## 1. Introduction

**Figure 1:**
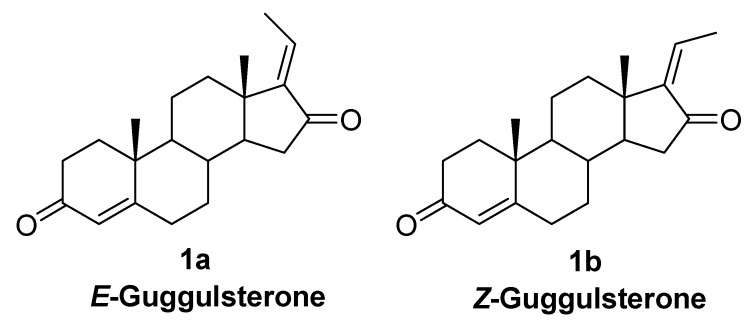
Chemical structures of *E*- and *Z*-guggulsterones.

Guggulipid from the resin of the *Commiphora mukul* tree (guggulu in Sanskrit) [1] has been used as an Asian folk remedy for chronic disorders such as rheumatism, obesity and atherosclerosis since at least 600 BC [2,3]. It has been reported that *E*- and *Z*-guggulsterones (Figure 1), two active ingredients in the *Commiphora mukul* resin [1,4] lower the level of low density lipoprotein cholesterol (LDLc) [5,6,7,8] and triglycerides in mouse. Guggulsterones are also known to have therapeutic effects for the treatment of inflammatory bowel diseases [9] and various cancers [10], and the molecular mechanisms underlying those effects are currently under investigation. Thus, there is a great demand for large amounts of the guggulsterones to further *in vitro and in vivo* studies. Because this demand has not been met by natural sources, which only provides the compounds in low yield (1.1%), [1,4] we have developed a first regioselective synthesis of guggulsterone.

In 1964, the first synthesis of guggulsterone was reported by Benn and Dodson [11,12], and a patent by Hamied and co-workers was published in 1991 [13]. In Benn and Dodson’s method, [11,12] the final *E*- and *Z*-guggulsterones were prepared from 16-dehydropregnenolone acetate (16-DPA) or 16,17-epoxypregnenolone (**2**) as starting steroid. Our initial attempts to synthesize guggulsterone were based on this protocol. However, the low yields and long reaction times ultimately led us to abandon this route. Moreover, we sought to investigate a stereoselective preparation of the guggulsterones because this was not detailed in either the papers or the patent.

## 2. Results and Discussion

**Scheme 1:**
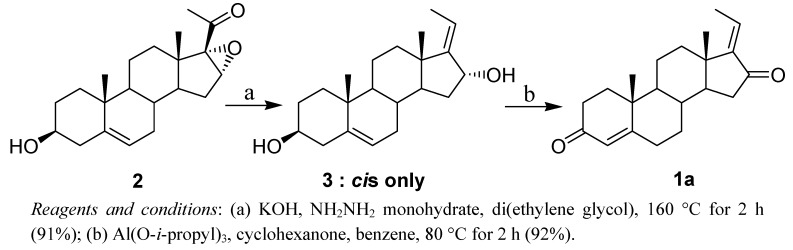
Regioselective synthesis of *E*-guggulsterone.

During the course of our synthetic studies of bioactive compounds, we discovered a stereoselective two-step reaction for the preparation of *E*-guggulsterone from 16,17-epoxypregnenolone (**2**) through a hydrazine reduction [11,14] and Oppenhauer oxidation [15,16]. Herein, we report a regioselective method for the preparation of *E*-guggulsterone and methods for the conversion of *E*-guggulsterone into the corresponding *Z* isomer (Scheme 1).

In the first step, we prepared the *cis*-diol **3** from steroid **2** using hydrazine monohydrate (98% purity) and 9.0 equiv of KOH at 160 °C for 2 h (91% yield). This stereoselectivity and yield differ dramatically from those reported in the literature for the same reaction. Specifically, Benn and Dodson reported that compound **3** was obtained as a mixture (66% *cis*-**3** and 34% *trans*-**3**) in 22% yield when using hydrazine at 195 °C for 5.5 h [11,14]. In 1968, Kessar and Rampal reported the preparation of *cis*-**3** using 80% hydrazine. They obtained a total yield of only 47% as a mixture of *cis*- and *trans*-**3** [14]. Based on these results, we identified two important factors that affect the regioselectivity and yield of *cis*-**3**: the purity of hydrazine monohydrate and the reaction time (reaction times greater than 3 h decrease the stereoselectivity). In the second and final step, the target compound, *E*-guggulsterone, was prepared through an Oppenhauer oxidation. We investigated the reaction conditions in order to maximize both regioselectivity and yield (Table 1).

**Table 1:** Optimization of Oppenhauer Oxidation Conditions for the Synthesis of *E*-Guggulsterone. *^a^* 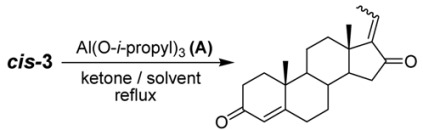

Entry	Equiv of A	Ketone	Solvent	Time (h)	% Yield *^b^*	*E:Z* Ratio *^c^*
1	0.5	cyclohexanone	toluene	2	94	67:33
2	1.0	cyclohexanone	toluene	2	96	86:14
**3**	**0.5**	**cyclohexanone**	**benzene**	**2**	**92**	**only *E***
4 *^d^*	1.0	acetone	benzene	7	no reaction	-
5	0.5	2-butanone	benzene	4	32	only *E*

*^a^* All reactions were carried out on a 1.0 mmol scale of *cis*-3; *^b^* The yields refer to the average isolated yield of three runs; *^c^E*: *Z* ratios were calculated based on ^1^H-NMR through the integration of peaks at 5.75 ppm and 6.52 ppm, respectively. *^d^* This reaction was performed in a sealed tube at 120 °C.

When the reaction was run in toluene using 0.5 equiv of Al(O-*i*-propyl)_3_, the desired compound was obtained as a mixture of isomers in 94% yield (Table 1, entry 1). By increasing the Al(O-i-propyl)_3_ loading from 0.5 equiv to 1.0 equiv, the regioselectivity for *E*-guggulsterone increased from 67% to 86% and the yield also increased slightly. The choice of solvent also proved to play an important role in the regioselectivity of the reaction; when benzene and 0.5 equiv of Al(O-i-propyl)_3_ were used, we obtained pure *E*-guggulsterone in 92% yield (Table 1, entry 3). On the other hand, when we changed the from cyclohexanone to acetone, the reaction did not proceed at all (Table 1, entry 4). When 2-butanone was used as an oxidant in the presence of 1.0 equiv of Al(O-*i*-propyl)_3 _at 80 °C for 4 h, *E*-guggulsterone was obtained as a single isomer in poor yield (Table 1, entry 5). Based on these results, we concluded that the most important variable for the preparation of pure *E*-guggulsterone was the reaction temperature. Next, we examined the isomerization of *E*-guggulsterone to *Z*-guggulsterone under various reaction conditions such as heat, light (hν), and acid catalysis. The results of the isomerization reactions are summarized in Table 2.

**Table 2:** Reaction Conditions for Isomerization from *E*- to *Z*-Guggulsterone. ^a^ 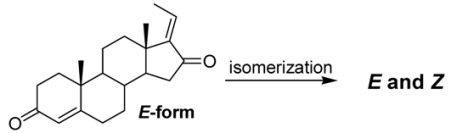

Entry	Driving force	Solvent/Temp.	Time (h)	*E(1a):Z(1b)* Ratio *^b^*
1	heat	toluene/110 °C	2	95:5
2	heat	mesitylene/170 °C	2	65:35
**3**	heat	toluene/sealed tube,140 °C	2	45:55
4	light *^c^*	MeOH/25 °C	12	50:50
5	*p-*TsOH	benzene/80 °C	1	40:60
6	2 *N*-HCl	acetonitrile/36 °C	18	60:40

^a^ All reactions were carried out on a 0.5 mmol scale; ^b^ The yield refer to the average isolated yield of three runs; ^c ^Light source was a 300 W-tungsten lamp.

Heating compound **1a **for 2 h at reflux (high temperature) gave 5-35% yields of the *Z*-isomer **1b**, (entries 1-2). When the reaction was carried out at 140 °C using a sealed tube, the yield of the *Z*-isomer was increased to 55% (Table 2, entry 3). In the light-induced isomerization (Table 2, entry 4), which contained 1.0 mol % of methylene blue as a photosensitizer, the E-isomer dissolved in CH_2_Cl_2_ was converted to the *Z*-isomer **1b** in the 50% yield after 12 h at 25 °C. On the other hand, the use of acid catalysts showed that conversion to *Z*-guggulsterone **1b** was higher when using *p*-toluenesulfonic acid as compared to 2N HCl, which is condition that mimics the human stomach (Table 2, entries 5 and 6). Interestingly, *E*- and *Z*- guggulsterones were very stable during the isomerization reaction and did not generate side products. They were also easily purified by chromatography on silica gel with hexane/EtOAc (v/v = 5/4).

## 3. Experimental

### 3.1. General

All reactions were performed in oven- and flame-dried glassware under nitrogen atmosphere. Air and moisture sensitive reagents and solvents were transferred *via* syringes or cannula, and they were introduced into the reaction vessel through a rubber septum. Chemicals obtained from commercial sources were used without further purification. Flash column chromatography was carried out on silica gel (230–400 mesh). Analytical thin-layer chromatography (TLC) was performed with silica gel 60 F254. TLC plates were visualized with UV light and 5% ammonium dimolybdate or *p*-anisaldehyde in ethanol with heat. NMR spectra (300 MHz for ^1^H and 75 MHz for ^13^C) were recorded in CDCl_3_ on a Bruker Avance III 400 MHz NMR spectrometer and chemical shifts (δ) were expressed in ppm downfield from the internal tetramethylsilane or with reference to residual CHCl_3_. The purity of compounds was assessed by HPLC/MS spectra, which were recorded on a Finnigan LTQ LC/MS system. Optical rotations were measured on a Rudolph Research Autopol Model III polarimeter.

*5,17(20)-(cis)-Pregnadiene-3β,16α-diol* (**3**): To a suspension of 16*α*,17*α*-epoxypregnenolone (3.31 g, 10.0 mmol) in diethylene glycol (25 mL, 99%) was added KOH (5.0 g, 89.0 mmol) and hydrazine monohydrate (9.7 mL, 200 mmol) at room temperature. After the mixture was heated at 120 °C for 1 h, the condenser was removed and the reaction temperature of 160 °C maintained for 2 h. The reaction was monitored by thin-layer chromatography. After being completely reacted, it was cooled, poured into water (30 mL) and extracted with CHCl_3_ (3 ° 40 mL). The combined organic layer was washed with brine, dried over MgSO_4_, filtered, and evaporated under reduced pressure to give the crude product. The crude compound was purified by recrystalization from hot ethyl acetate to obtain **3** as a white solid (2.88 g, 91%). ^1^H-NMR: *δ* 5.60 (q, 1H, *J* = 7.1 Hz), 5.36 (d, 1H, *J* = 5.3 Hz), 4.44 (s, 1H), 3.54 (m, 1H), 2.30−0.91 (m, 19H), 1.74 (d, 3H, *J* = 7.1 Hz), 1.03 (s, 3H), 0.89 (s, 3H); ^13^C-NMR: *δ* 155.7, 141.2, 121.9, 120.0, 74.8, 72.1, 53.1, 50.5, 44.6, 42.7, 37.6, 37.5, 37.0, 35.6, 32.0, 31.2, 21.5, 19.8, 17.7, 13.7; mp = 192−194 °C; [α]_D_ −79.2° (c=1.0, EtOH); HRMS (EI): calcd for C_21_H_32_O_2_ 316.2402, found 316.2402.

*E-Guggulsterone* (**1a**): To a suspension of **3** (2.0 g, 6.3 mmol) in benzene (60 mL) was added cyclohexanone (6.6 mL, 63.0 mmol), followed by addition of Al(O-isopropyl)_3 _(650 mg, 3.2 mmol) at the room temperature. The reaction mixture was warmed at 80 °C for 2 h. After that, the mixture was cooled to the room temperature, added 10% H_2_SO_4_ (4 mL), and vigorously stirred for 10 min. The organic layer was separated and the aqueous layer was extracted with ethyl acetate (2 ° 35 mL). The combined extract was washed with water, dried over anhydrous MgSO_4_, filtered, and evaporated under reduced pressure to give the crude product. The crude compound was purified by column chromatography on silica gel using hexane/ethyl acetate (v/v = 5/4) as eluent to give **1a** as a white solid (1.62 g, 91%). ^1^H-NMR: *δ* 6.52 (q, 1H, *J* = 7.5 Hz), 5.75 (s, 1H), 2.50−1.08 (m, 19H), 1.86 (d, 3H, *J* = 7.5 Hz), 1.24 (s, 3H), 1.08 (s, 3H); ^13^C-NMR: *δ* 206.0, 199.6, 170.6, 147.8, 129.9, 124.5, 53.8, 49.9, 43.5, 39.0, 38.2, 36.4, 35.9, 34.7, 34.3, 32.9, 32.2, 21.1, 17.9, 17.7, 13.6; mp = 168−171 °C; [α]_D_ −34.5° (c=1.0, EtOH); HRMS (FAB): calcd for C_21_H_28_O_2_ [M+H]^+^ 313.4601, found 313.2168.

*Z-Guggulsterone* (**1b**): *(a) Photoreaction method*: Methylene blue (1 mg) was added as a photosensitizer to a solution of **1a** (1.0 g, 3.2 mmol) in CH_2_Cl_2_ (50 mL) at room temperature. The mixture was irradiated with 300W-tungsten lamp in water bath for 6 h. After that, solvent was removed under reduced pressure at the room temperature. The residue was purified by chromatography on silica gel with hexane/ethyl acetate (v/v = 5/4) to afford **1b** as a white solid (433 mg, 43%).^1^H-NMR: *δ* 5.73 (s and m, 2H), 2.43−0.75 (m,19H), 2.08 (d, 3H), 1.22 (s, 3H), 0.96 (s, 3H); ^13^C-NMR: *δ* 208.2, 199.6, 170.7, 148.2, 130.9, 124.5, 54.0, 49.4, 43.4, 39.7, 39.1, 35.9, 35.0, 34.3, 33.0, 32.2, 21.0, 19.9, 17.7, 14.5;mp = 191−193 °C; [α]_D_ −54.8° (c=1.0, EtOH); HRMS (FAB): calcd for C_21_H_28_O_2_ [M+H]^+^ 313.4601, found 313.2168. *(b) Sealed-tube method*: In a dried sealed tube, **1a **(200 mg, 0.64 mmol) was dissolved in toluene (20 mL) and then the tube was completely sealed with flame. The mixture was reacted at 160 °C for 2 h. After cooling to room temperature, solvent was removed under reduced pressure, and the residue was purified by chromatography on silica gel with hexane/ethyl acetate (v/v = 5/4) to obtain **1b** as a white solid (117 mg, 59%) and recovered **1a **(81 mg). *(c) Acid-catalyzed method*: To a solution of **1a** (1.0 g, 3.2 mmol) in benzene (50 mL) was added *p*–toluenesulfonic acid (61.0 mg, 0.32 mmol) at the room temperature. The resulting mixture was heated at 80 °C for 1 h. After that, the reaction mixture was cooled to room temperature and solvent was removed under reduced pressure. The residue was purified by chromatography on silica gel with hexane/ethyl acetate (v/v = 5/4) to obtain **1b** as a white solid (644 mg, 64%) and recovered **1a **(350 mg).

## 4. Conclusions

In conclusion, we have successfully prepared *E*-guggulsterone in 84% yield over two steps from 16,17-epoxy-pregnenolone via hydrazine reduction and Oppenhauer oxidation. Additionally, by using heat, light (*hν*), and acid catalysts to induce isomerization, we also easily converted *E*-guggulsterone into its corresponding *Z* isomer.
